# Porphyrin synthesis from ALA derivatives for photodynamic therapy. *In vitro* and *in vivo* studies

**DOI:** 10.1038/sj.bjc.6601722

**Published:** 2004-03-16

**Authors:** C Perotti, H Fukuda, G DiVenosa, A J MacRobert, A Batlle, A Casas

**Affiliations:** 1 Centro de Investigaciones sobre Porfirinas y Porfirias (CIPYP) FCEyN (University of Buenos Aires and CONICET), Ciudad Universitaria, Pabellón II, 2do piso, (1428) Capital Federal, Argentina; 2 National Medical Laser Centre, Royal Free and University College Medical School, London, UK

**Keywords:** photodynamic therapy, PDT, aminolevulinic acid, ALA, ALA derivatives

## Abstract

The aim of this work was to test in *vitro and in vivo* the efficacy of the derivatives of 5-aminolevulinic acid (ALA): hexyl-ALA (He-ALA), undecanoyl-ALA and *R*,*S*-2-(hydroximethyl)tetrahydropyranyl-ALA (THP-ALA) as pro-photosensitising agents. The compounds were assayed in a cell line derived from a murine mammary tumour, in tumour explants and after injection of the cells into mice. *In vitro*, undecanoyl-ALA and THP-ALA did not improve ALA efficacy in terms of porphyrin synthesis. On the other hand, half of the amount of ALA is required to obtain the same plateau amount of photosensitiser from He-ALA. However, this plateau value cannot be surpassed in spite of the four-times higher accumulation of ALA/He-ALA from the ALA derivative. This shows that He-ALA conversion to porphyrins but not He-ALA entry to the cells is limiting. Employing ionic exchange chromatography, we found that 80% of total uptake was He-ALA whereas only 20% was ALA. This suggests that the esterases, probably themselves regulated by the heme pathway, are limiting the conversion of ALA derivatives into porphyrins. A similar situation occurs with THP-ALA. Tumour explant porphyrin results correlate well with cell line data. However, i.p. injection of ALA derivatives to mice resulted in a lower porphyrin concentration in the tumour when compared to the administration of equimolar amounts of ALA, indicating that there should be retention of ALA derivatives either within the blood vessels in the initial phase of distribution and/or within the capillaries of the tumour.

5-Aminolevulinic acid (ALA) is the first intermediate in heme biosynthesis and a precursor of protoporphyrin IX (PpIX). Protoporphyrin IX is used as an endogenous photosensitiser in photodynamic therapy (PDT) (Kennedy *et al*, 1990). The main advantage of using PpIX relative to other photosensitisers is the short half life of its photosensitising effects, which do not last longer than 48 h (Fukuda *et al*, 1992). 5-Aminolevulinic acid-induced porphyrin fluorescence may also assist in the early detection of some malignancies (Baumgartner *et al*, 1996).

The hydrophilic nature of the ALA molecule appears to limit its penetration through the *stratum corneum* of the skin. Hence, ALA-induced PpIX formation is often restricted to superficial tissue layers because of both uneven and partial tissue distribution in the deeper-lying or nodular lessions (Peng *et al*, 1995). More lipophilic derivatives of ALA were expected to have better diffusing properties, and, after their conversion into the parent ALA by enzymatic hydrolysis, to reach a higher PpIX formation rate.

To induce higher PpIX formation and photosensitisation, several chemical modifications both on the amino and carboxyl groups of ALA have been made, from which different degrees of photosensitisation were achieved (Casas and Batlle, 2002).

Factors involved in the uptake of the pro-drugs and release of ALA are manifold: (a) diffusion rates through the tissues; (b) uptake by the cell; and (c) liberation of ALA from the derivatives. Owing to the multiple factors involved in final ALA availability, differences between *in vivo* and *in vitro* efficiencies from the different compounds used as pro-photosensitisers were expected (Casas *et al*, 2001a, 2001b; Perotti *et al*, 2002).

The aim of this work was to test in *vitro* and *in vivo* the efficacy of the following ALA derivatives as pro-photosensitisers: hexyl-ALA (He-ALA), undecanoyl-ALA and *R*,*S*-2-(hydroximethyl)tetrahydropyranyl-ALA (THP-ALA) in comparison with ALA in a murine mammary tumour.

## MATERIALS AND METHODS

### Cell line and cell culture

Cell line LM3 (Werbajh *et al*, 1998) derived from the murine mammary adenocarcinoma M3 was cultured in minimum essential Eagle's medium, supplemented with 2 mM L-glutamine, 40 *μ*g gentamycin ml^−1^ and 5% foetal bovine serum (FBS), and incubated at 37°C in an atmosphere containing 5% CO_2_. Cells were used 48 h after plating.

### Chemicals

5-Aminolevulinic acid was from Sigma Chemical Co. St. Louis, MO, USA 5-Aminolevulinic acid derivatives were obtained as the hydrochloric salts. Hexyl-ALA and undecanoyl-ALA were synthesised according to the method of Takeya (1992), by reacting ALA with hexanol and undecanol, respectively, in the presence of thionyl chloride. The mixture was stirred at 70°C until ALA·HCl was completely dissolved, and the reaction was confirmed by TLC (Cl_2_CH_3_ : MeOH 9 : 1). The excess alcohol was evaporated under high vacuum. After addition of diethylether, the HCl salts of the ALA esters were allowed to crystallise at 4°C. Yields ranged from 40 to 60%. *R*,*S*-ALA-2-(hydroximethyl) tetrahydropyranyl ester was similarly obtained. The crude product was purified by flash column cromatography on silica gel eluting with Cl_2_CH_3_/MeOH mixtures. The yield was 20%. Purity of the synthesised compounds was always higher than 95%, as established by TLC and NMR techniques.

5-Aminolevulinic acid derivatives were dissolved in phosphate buffer saline (PBS) immediately before use, and the resulting solutions were sterilised by filtration through 0.21 *μ*m pore size filters. Addition of either ALA or ALA derivatives to the cells did not change the pH of the medium.

### Porphyrin extraction from cells

Porphyrins accumulated within the cells were extracted twice with 5% HCl, leaving the cells standing for half an hour in the presence of the acid at 37°C. For media determinations, 5% HCl was added and measured directly. These conditions proved to be the optimal for total PpIX extraction. Excitation and emission wavelengths of light producing the highest fluorescence were used (406 and 604 nm, respectively). Protoporphyrin IX (Porphyrin Products, Logan, UT, USA) was used as a reference standard.

### 5-Aminolevulinic acid and porphobilinogen (PBG) determination in cells

We have previously found that THP-ALA, undecanoyl-ALA and He-ALA content can be determined indistinguishably from ALA by the method of Di Venosa *et al* (2003). Cells were seeded in 100 mm dishes. After 48 h, medium was removed and cells were exposed for 3 h to 0.6 mM ALA or ALA derivatives in serum-free medium. Afterwards, cells were washed four times with PBS and then 5% TCA was added. After scrapping, the cells were centrifuged and the supernatant was employed for ALA and PBG determination following the method of Mauzerall and Granick (1956), slightly modified as indicated. Briefly, for ALA determination, a condensation reaction was developed in the presence of acetyl acetone and the resulting pyrroles were quantified by addition of the Ehrlich's reagent. 5-Aminolevulinic acid and ALA derivative standards were condensed under similar conditions and thereafter employed for calculations. For PBG determination, the Ehrlich's reagent was added to the deproteinised TCA supernatant. 5-Aminolevulinic acid values were obtained by subtracting PBG values from the total value of condensed pyrroles.

### 5-Aminolevulinic acid, He-ALA and THP-ALA separation using ion-exchange column chromatography

Cells were plated in 100 mm dishes. A pool of two dishes per point was used. After 72 h, the medium was removed and cells were exposed for 3 h to 0.6 mM ALA or ALA derivatives in serum-free medium. Afterwards, cells were washed four times with PBS and 5% TCA was added. After scrapping, the cells were centrifuged and the content of two dishes was pooled. An aliquot of the each supernatant was employed for ALA and PBG determination, as described above. The rest of the supernatant was passed through a column of Dowex 50 X 8 resin, as reported elsewhere (Di Venosa *et al*, 2003). 5-Aminolevulinic acid was separated from ALA derivatives, and the percentage of the total was calculated from the total ALA after subtracting the PBG value.

### Photodynamic therapy treatment *in vitro*

Cells seeded in six-well plates were incubated in serum-free medium containing ALA or ALA derivatives; 3 h later, irradiations were performed. After irradiation, the medium was replaced by ALA-free medium+FBS, the cells were incubated for another 19 h and then tested for viability.

### Light source

For PDT experiments, a bank of two fluorescent lamps (Osram L 36W/10) was used. The spectrum of light was between 400 and 700 nm, with the highest radiant power at 600 nm. The plates were located at a distance of 60 cm from the light source. Fluence rate was measured with a Yellow Springs Kettering model 65 radiometer (Yellow Springs, OH, USA).

### MTT viability assay

Phototoxicity and cell viability were documented by the MTT assay (Denizot and Lang, 1986), a method based on the activity of mitochondrial dehydrogenases, which will be functionally affected by PDT *in vitro* (Hilf *et al*, 1984). Following appropriate treatments, 3-[4,5-dimethylthiazol-2-yl]-2,5-diphenyltetrazoliumbromide (MTT) solution was added to each well in a concentration of 0.5 mg ml^−1^, and plates were incubated at 37°C for 1 h. The resulting formazan crystals were dissolved by the addition of DMSO and absorbance was read at 560 nm.

### Animals

Male BALB/*c* mice, 12 weeks old, weighing 20–25 g were used. They were provided with food (Purina 3, Molinos Rio de la Plata) and water *ad libitum*. A suspension of 1.65 × 10^5^ cells of the LM3 cell line was subcutaneously injected on the flanks of male BALB/*c* mice. Experiments were performed at approximately day 20 after implantation. Tumours of the same uniform size were employed (1 cm diameter). Animals received human care and were treated in accordance with the guidelines established by the Animal Care and Use Committee of the Argentine Association of Specialists in Laboratory Animals (AADEALC), in full accord with the UK Guidelines for the Welfare of animals in Experimental Neoplasia (Workman *et al*, 1998).

### 5-Aminolevulinic acid administration

The hydrochloric salt of ALA and ALA derivatives was dissolved in saline in a final volume of 0.15 ml immediately before intraperitoneal (i.p.) injection.

Times of killing were chosen according to optimal ALA and He-ALA conditions (Perotti *et al*, 2002), with the aim of correlating ALA with porphyrin content at a certain time point.

### Tumour porphyrin extraction

After ALA or ALA derivative injection, animals were killed. Before killing, mice were injected with heparin (0.15 ml, 1000 UI) and after killing they were perfused with 200 ml of sterile saline. The tumour samples were homogenised in a 4 : 1 solution of ethyl acetate : glacial acetic acid mixture according to Batlle (1997). Briefly, the mixtures were centrifuged for 30 min at 3000 **g**, and the supernatants were added with an equal volume of 5% HCl. Extraction with HCl was repeated until negative fluorescence in the organic layer. The aqueous fraction was used for the determination of porphyrins. For fluorometric determination, a Shimadzu RF-510 spectrofluorometer was used, with an emission wavelength at 604 nm and an excitation wavelength at 406 nm, employing PpIX as a reference standard.

### 5-Aminolevulinic acid and PBG determination in tumour tissue

Briefly, 100 mg of tissue were homogenised in 1 M acetic acetate buffer (pH 4.8); 5% TCA was added to deproteinise and samples were centrifuged for 30 min at 3000 **g**. 5-Aminolevulinic acid and PBG content were determined as described above, except for a second centrifugation that was performed after ALA condensation.

### Organ explant cultures

The explant tissue culture system developed by Polo *et al* (1988) was used. Tumour explants of about 50 mg were floated in petri dishes in serum-free minimal essential Eagle's medium, supplemented with 2 mM L-glutamine and gentamycin (40 *μ*g ml^−1^) and incubated at 37°C in presence of 0.6 mM ALA or ALA derivatives for 3 h, washed with PBS and processed for porphyrin extraction, as described above. The optimal conditions for incubation and explant sizes were as determined in a previous work (Fukuda *et al*, 1989).

### Statistical analysis

The unpaired *t*-test was used to establish the significance of differences between groups. Differences were considered statistically significant when *P*<0.05*. In vitro* experiments: three independent experiments, in triplicates. *In vivo* experiments: three mice per group were employed.

## RESULTS

Figure 1

**Figure 1:**
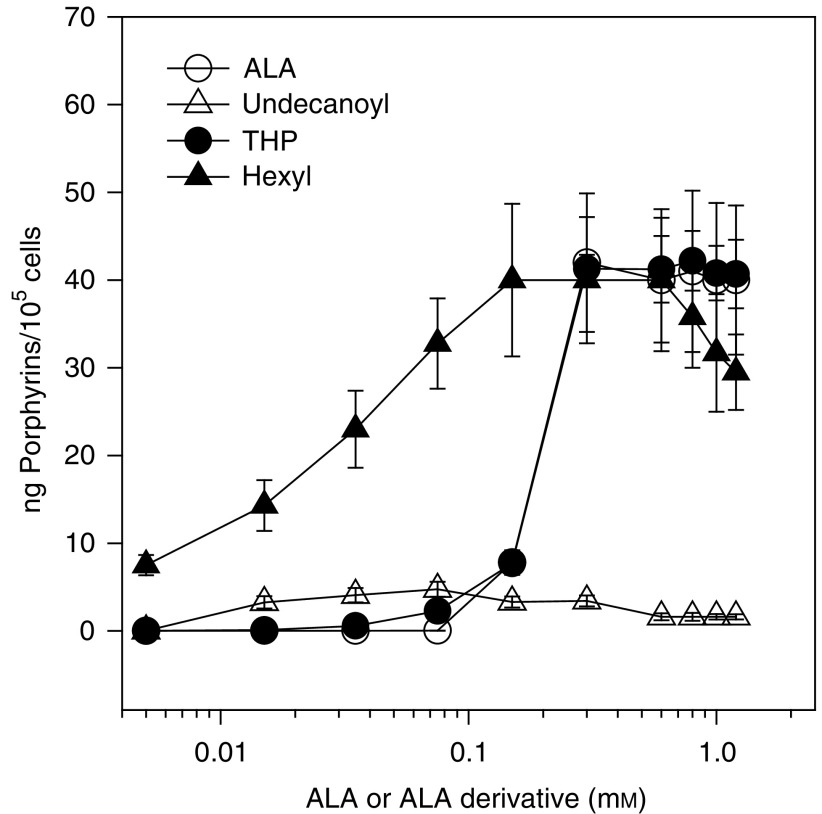
Porphyrin synthesis from ALA and ALA derivatives. Cells were incubated for 3 h in the presence of different amounts of ALA or its derivatives. Intracellular porphyrins were determined fluorometrically and relativised per number of cells present at the beginning of the experiment.

shows the dependance of porphyrin synthesis on ALA or ALA derivative concentration. We find saturation points at 0.3 mM ALA and THP-ALA, 0.15 mM He-ALA and 0.075 mM undecanoyl-ALA. The maxima porphyrin synthesis from ALA, THP-ALA and He-ALA is around 40 *μ*g/10^5^ cells. Undecanoyl-ALA only produces 4.5 *μ*g/10^5^ cells under the best conditions. Porphyrin synthesis from ALA and THP-ALA exhibits a sigmoidal shape, showing a sharp increase in porphyrin synthesis at 0.1 mM ALA. On the contrary, porphyrin synthesis increases gradually upon increasing the hexyl and undecanoyl derivative concentration.

**Figure 2:**
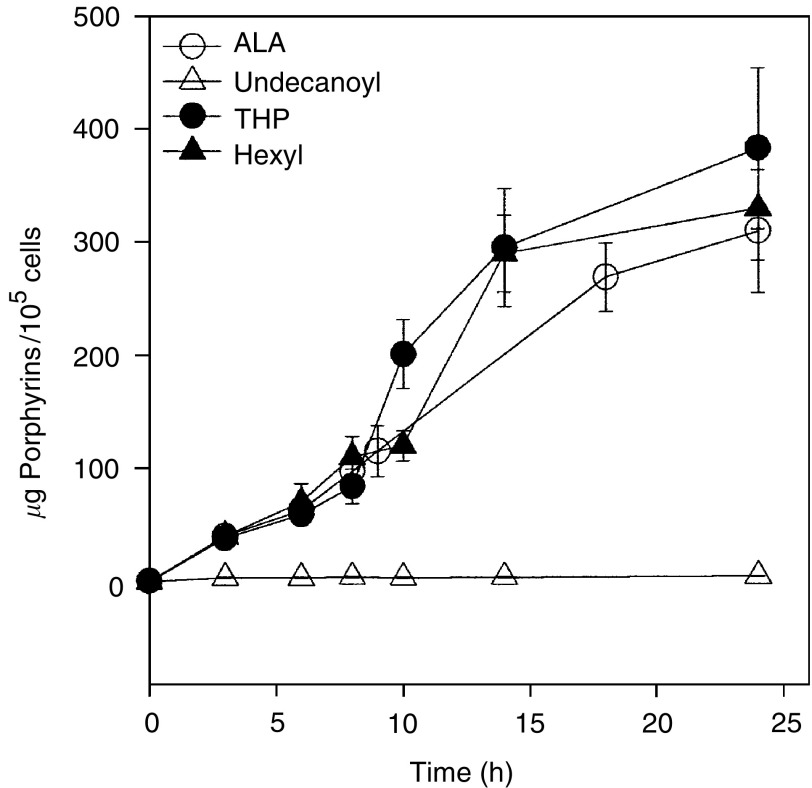
Porphyrin synthesis as a function of incubation time in the presence of ALA and derivatives. Cells were incubated during different time periods in the presence of 1.2 mM ALA, THP-ALA and He-ALA and 0.3 mM undecanoyl-ALA in 24-well plates. Intracellular porphyrins were determined fluorometrically and relativised per number of cells present at the beginning of the experiment.

The time dependance of porphyrin synthesis in the presence of ALA and ALA derivatives under saturating conditions is shown in Figure 2

. An almost linear increase is shown for porphyrin synthesised from ALA, He-ALA and THP-ALA. A low amount of porphyrins is formed from 0.3 mM undecanoyl-ALA, and this concentration could not be further increased due to intrinsic toxicity at longer incubation times.

Figure 3

**Figure 3:**
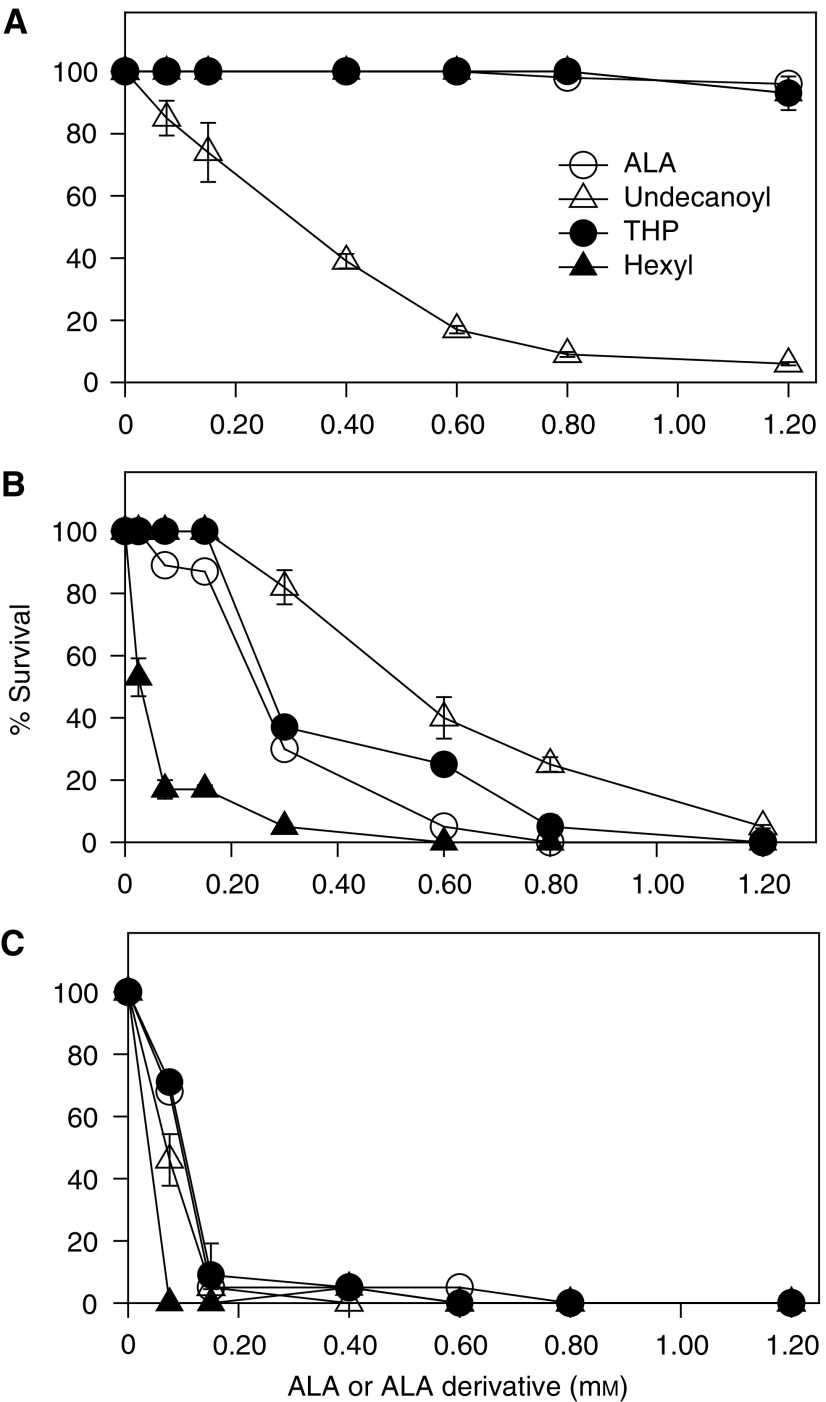
Dark and PDT toxicity of ALA and ALA derivatives. Cells were incubated for 3 h in the presence of different amounts of ALA or its derivatives in the dark (**A**) or exposed to 0.25 (**B**) and 0.4 J cm^−2^ of light (**C**). The MTT assay was performed after 19 h. Cell survival is expressed as a percentage of the control nonirradiated and exposed to ALA or derivatives.

depicts dark toxicity and PDT toxicity, varying with ALA or ALA derivative concentration. The only compound that is intrinsically toxic to the cells without light is undecanoyl-ALA, which is harmful at very low concentrations. At low doses, He-ALA is more effective than ALA due to the threshold required for porphyrin synthesis.

Figure 4

**Figure 4:**
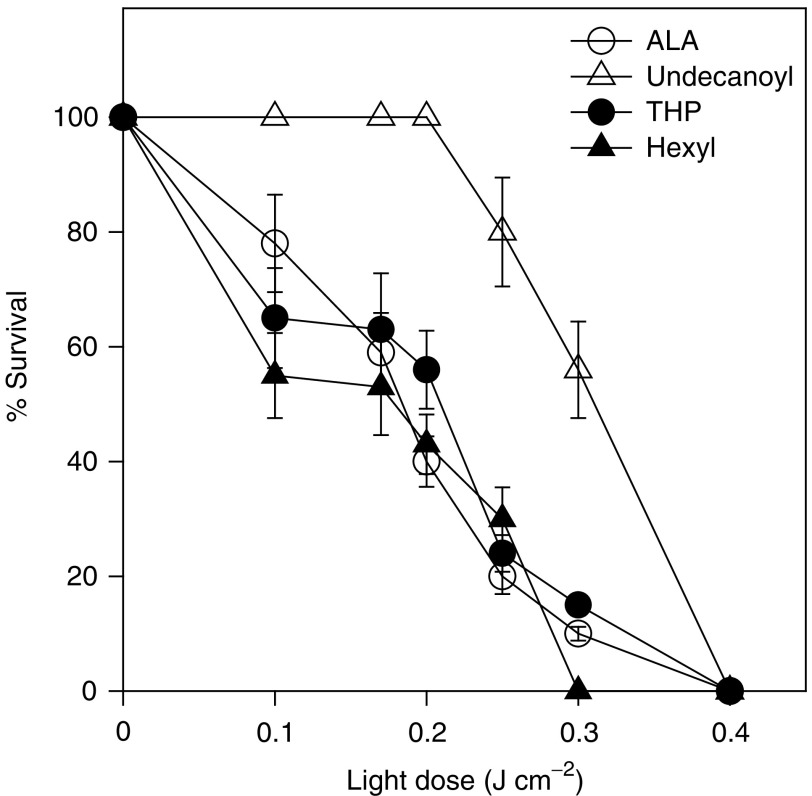
Photodynamic therapy with ALA or ALA derivatives and the dependance on light dose. Cells were incubated for 3 h in the presence of a fixed concentration of ALA (0.6 mM), undecanoyl-ALA (0.3 mM), THP-ALA (0.6 mM) or He-ALA (0.6 mM) and exposed to a varying light dose. The MTT assay was performed after 19 h. Cell survival is expressed as a percentage of the control nonirradiated and exposed to ALA or derivatives.

shows the PDT response driven by ALA and its derivatives, varying with the light dose. The ALA and ALA derivative concentrations employed produced maximal porphyrin synthesis. We can observe that on increasing the light dose PDT toxicity increases similarly for ALA, THP-ALA and He-ALA, whereas for undecanoyl-ALA, a 40% decrease of viability is observed at 0.3 mM and concentrations above 0.3 mM lead to complete cell death, but due to intrinsic toxicity of the derivative and not to the PDT effect.

Table 1

**Table 1:** ALA and PBG accumulation in cells

	**pmol ALA or ALA derivatives/10^5^ cells^a^**	**ALA (percentage of ALA+ALA derivative)^b^**	**pmol PBG/10^5^ cells**
Control	1.62±0.07	ND	0.75±0.03
ALA	13.05±0.90	100%	1.5±0.11
He-ALA^a^	42.41±6.20	20%	1.7±1.3
Undecanoyl-ALA	3.15±0.12	ND	0.8±0.04
THP-ALA	36.3±4.78	40%	2.07±0.46

ALA and PBG were determined as described in Materials and methods after 3 h exposure to 0.6 mM ALA or ALA derivatives.

a ALA determinations correspond to the ALA+ALA derivative intracellularly accumulated in the cells, without distinction between ALA and its derivative. Controls correspond to basal levels of untreated cells. ND=nondetectable.

b Determined by ionic exchange chromatography.

shows ALA and PBG accumulation in cells treated with ALA or ALA derivatives. We can clearly see that ALA and/or He-ALA accumulated from He-ALA is four times higher than ALA accumulated from ALA. However, PBG accumulation is not higher from He-ALA, and neither is porphyrin synthesis (see Figure 1) due to the low conversion of He-ALA into ALA (only 20%). The amount of ALA and/or THP-ALA accumulated from THP-ALA is 2.8 times higher than the amount formed by ALA, but only 40% is converted into ALA, so that again PBG and porphyrin synthesis remain the same. ALA and/or undecanoyl-ALA accumulated from the former is very low and could not be separated by chromatography.

Table 2

**Table 2:** Porphyrin synthesis in tumour explants incubated with ALA and ALA derivatives

	***μ*g porphyrin/g tissue**
Control	0.33±0.02
ALA	5.17±0.26
He-ALA	5.83±0.38
Undecanoyl-ALA	1.18±0.07
THP-ALA	5.53±0.27

Tumour explants were incubated for 3 h with 0.6 mM ALA or ALA derivatives. Porphyrins were extracted and determined as described in Materials and Methods. Controls correspond to the tissue basal porphyrin levels.

shows tumour explant porphyrin synthesis from 0.6 mM ALA and ALA derivatives. Porphyrin synthesis from He-ALA and THP-ALA is similar to that produced from ALA. Undecanoyl-ALA porphyrin synthesis was five-fold lower, hardly above the basal levels.

Table 3

**Table 3:** Tumour porphyrin synthesis from ALA and ALA derivatives after systemic administration to mice and ALA content

	**nmol ALA or ALA derivatives/g tissue at 10 min**	**nmol ALA or ALA derivatives/g tissue at 3 h**	**nmol porphyrins/g tissue at 3 h**
Control	12.5±0.9	12.5±0.9	0.36±0.05
ALA	170.2±8.4	15.11±5.1	15.9±1.12
He-ALA	56.3±10.3	12.9±1.5	3.11±0.42
Undecanoyl-ALA	28.2±3.1	11.2±3.4	2.64±0.23
THP-ALA	37.0±5.2	17.8±2.8	8.83±0.95

In all, 3.3 mg ALA, 5 mg He-ALA, 6.3 mg undecanoyl-ALA and 5.2 mg THP-ALA were administrated i.p. to mice. After 10 min, the ALA content was determined and after 3 h ALA and porphyrins were quantified in tumour. PBG levels at 10 min and 3 h were undetectable.

shows tumour porphyrin synthesis after systemic administration of equimolar concentrations of ALA and ALA derivatives to mice. 5-Aminolevulinic acid is the best inducer of porphyrin synthesis under these conditions, followed by THP-ALA, He-ALA and undecanoyl-ALA. A much higher amount of ALA than ALA derivatives reaches the tumour 10 min after i.p. administration. After 3 h, the same amount of ALA is retained within the tissue from ALA and from ALA derivatives.

## DISCUSSION

I.p. injection to mice of the derivatives He-ALA and undecanoyl-ALA resulted in porphyrin concentrations five-fold lower in tumour when compared to equimolar amounts of ALA administration. THP-ALA induced half the amount of porphyrins produced by ALA. Concentration of the derivatives could not be increased further because they were lethal to mice.

When the LM3 cell line was exposed to undecanoyl-ALA, we have found that toxicity was actually due to the release of the alcohol after the esterase action (data not shown). However, *in vivo* toxicity of He-ALA and undecanoyl-ALA may be provoked by a different mechanism. When higher doses of He-ALA and undecanoyl-ALA are used, mice die suddenly within the following 5 min. Based on the observation that He-ALA induces a high porphyrin synthesis in brain (Perotti *et al*, 2002), we had suspected that He-ALA was neurotoxic; however, this cannot be applied to undecanoyl-ALA because this ALA derivative produces negligible amounts of porphyrins in brain (Casas *et al*, unpublished results).

Porphyrin synthesis from He-ALA and undecanoyl-ALA *in vitro* increases linearly from the beginning of the concentration curves. Instead, ALA and THP-ALA do not induce porphyrin synthesis up to 0.1 mM. This suggests that their incorporation mechanisms into the cell are different, and that, whereas ALA and THP-ALA uptake appear to need a threshold concentration, He-ALA uptake takes place even at low concentrations. It has been shown that in LM3 cells ALA uptake occurs by both diffusion and active transport, the latter presumably mediated by a member of the GAT family transporters (Bermúdez Moretti *et al*, 2002). Here, we have evidence that He-ALA and undecanoyl-ALA are incorporated by a different transport system. Krieg *et al* (2002) studied the kinetics of PpIX after exposure of human colonic cell lines and fibroblasts to He-ALA and benzyl-ALA. They also found that detectable amounts of PpIX already synthesised from these ALA esters were formed much earlier than from ALA, showing that passive diffusion happens much faster than active transport of ALA via the beta transporters.

Similarly, from kinetic studies, Whitaker *et al* (2000) hypothesised that pentyl-ALA is incorporated into pancreatoma cells at a faster rate than ALA, indicating that esterases are not limiting porphyrin synthesis and that the ester must be translocated by rapid diffusion. Depending on the pro-drug, different transport mechanisms would be operating to reach more quickly the threshold ALA level required for inducing porphyrin biosynthesis.

It has been previously reported that long-chained ALA esters tend to remain in the cell membrane, thus synthesising lower PpIX (Kloek *et al*, 1998). However, we have found that in cells low concentrations of undecanoyl-ALA (up to 0.1 mM) lead to higher porphyrin synthesis than that produced from ALA, although this amount cannot be increased by increasing the ALA-ester concentration above 0.1 mM.

Eléouet *et al* (2000) found that rat glial tumour cells exposed to THP-ALA were more efficiently photosensitised than cells exposed to equimolar ALA concentrations. The same authors have shown that THP-ALA sensitised the cells at concentrations as low as 18 *μ*M. However, in our cells, THP-ALA was not found to be a better pro-photosensitiser than ALA. We ascribe these differences in PDT response to the different cell types. We might speculate that different ALA esters may be incorporated into the cells by different transport mechanisms and that these mechanisms may vary for each cell type.

Half of the amount of ALA is required to obtain the same plateau amount of photosensitiser from He-ALA. However, this plateau value cannot be surpassed in spite of the four-times higher accumulation of ALA/He-ALA from the ALA derivative. This shows that He-ALA conversion to porphyrins but not He-ALA entry to the cells is limiting. To test whether or not esterase converts He-ALA into ALA, we separated ALA from He-ALA by ionic exchange chromatography. We found that 80% of the total compound was He-ALA, whereas only 20% was ALA. This means that around 8.5 pmol of ALA is available for porphyrin synthesis, and that esterases are limiting ALA conversion into porphyrins. A similar situation occurs with THP-ALA. Although 2.8 times more ALA/THP-ALA is accumulated in the cells, only 40% is converted into ALA. In other words, only 14 pmol of ALA is available for porphyrin synthesis. This might be linked to some kind of regulation through the esterases, which would provide just the amount of ALA to be employed for porphyrin synthesis, probably by means of a product regulation. In this regard, we could speculate that esterases could be ultimately be regulated by the heme pathway. However, more data are needed to confirm this hypothesis.

5-Aminolevulinic acid/undecanoyl-ALA accumulation from undecanoyl-ALA is very low and could not be separated by chromatographic techniques, but in this case there is a good correlation between ALA accumulation and porphyrin synthesis, apparently being under nonsaturating conditions of the esterases.

*In vitro* experiments with tumour explants are keeping in line with cell data. Employing 0.6 mM of ALA and ALA esters, porphyrin synthesis from ALA, He-ALA and THP-ALA is similar, while porphyrin accumulation from undecanoyl-ALA is hardly above basal levels. These results indicate a good correlation between both *in vitro* models, which are markedly different from *in vivo* data.

Hexyl-ALA has previously proved to considerably induce PpIX synthesis *in vitro* but not *in vivo.* These surprisingly huge differences between results obtained with cell lines and their parental tumours may be ascribed to a large number of factors: (a) diminished ability of He-ALA for crossing vascular structures to reach tumoural cells, (b) retention of He-ALA in the interstitial space and consequent limited availability to cell layers, (c) differential expression and activity of esterases in the cell line as compared to the parental tumour (Casas and Batlle, 2002).

Around 170 nmol ALA g^−1^ tissue is retained in the tumour after ALA injection, whereas three to five times less ALA is accumulated from ALA derivatives. However, after 3 h the ALA concentration is similar from all compounds. Although three times less ALA is accumulated from He-ALA, when compared to ALA porphyrin synthesis is five times lower; however, the rest of ALA is not present at 3 h. Distribution of He-ALA to other tissues is possible.

Protoporphyrin IX synthesis *in vitro* does not reflect at all the efficiency found *in vivo*, neither ALA nor ALA derivative accumulation *in vitro* correlates with *in vivo* data, demonstrating the complexity and difficulty for extrapolating results from *in vitro* to *in vivo* models. The data suggest that after ALA or ALA ester i.p. injection, retention of ALA derivatives either within the blood vessels in the initial phase of distribution or/and within the capillaries of the tumour is playing an important role in the availability of ALA.

It is clear from the state of the art that the relevance of using ALA derivatives lies in the possibility of decreasing ALA derivative concentrations to reach equal porphyrin synthesis, and gaining selectivity, without enhancing ALA accumulation. Our findings underline the importance of working with ALA derivatives under nonsaturating conditions of the esterase activities and/or heme pathway. Further studies on the esterase saturation points and their regulation will be the subject of future work.
